# The Relative Contribution of Glycine–GABA Cotransmission in the Core of the Respiratory Network

**DOI:** 10.3390/ijms25063128

**Published:** 2024-03-08

**Authors:** Ali Harb, Charlotte Tacke, Behnam Vafadari, Swen Hülsmann

**Affiliations:** Department of Anesthesiology, University Medical Center, Georg-August University, 37099 Göttingen, Germany

**Keywords:** neurotransmitters, vesicular filling, synaptic structure, synaptic transmission, synaptic function, neurotransmitter release

## Abstract

The preBötzinger complex (preBötC) and the Bötzinger complex (BötC) are interconnected neural circuits that are involved in the regulation of breathing in mammals. Fast inhibitory neurotransmission is known to play an important role in the interaction of these two regions. Moreover, the corelease of glycine and GABA has been described in the respiratory network, but the contribution of the individual neurotransmitter in different pathways remains elusive. In sagittal brainstem slices of neonatal mice, we employed a laser point illumination system to activate glycinergic neurons expressing channelrhodopsin-2 (ChR2). This approach allowed us to discern the contribution of glycine and GABA to postsynaptic currents of individual whole-cell clamped neurons in the preBötC and BötC through the application of glycine and GABA receptor-specific antagonists. In more than 90% of the recordings, both transmitters contributed to the evoked IPSCs, with the glycinergic component being larger than the GABAergic component. The GABAergic component appeared to be most prominent when stimulation and recording were both performed within the preBötC. Taken together, our data suggest that GABA–glycine cotransmission is the default mode in the respiratory network of neonatal mice with regional differences that may be important in tuning the network activity.

## 1. Introduction

Breathing depends on coordinated and rhythmic neuronal activity generated by a network of neurons in the brainstem. The network’s core is located bilaterally in the ventral respiratory column (VRC), which comprises three regions: the Bötzinger complex (BötC), preBötzinger complex (preBötC), and rostral ventral respiratory group (rVRG) [1]. While glutamatergic neurons in the preBötC generate the inspiratory drive of the network [2,3,4], mutually inhibitory interactions between the BötC and preBötC are discussed to orchestrate the alternating phases of breathing, with high activity levels of BötC neurons during the expiratory, or post-inspiratory, phase [5,6,7].

Since GABA and glycine require the same vesicular inhibitory amino acid transporter (VIAAT, SLC32A1) for loading, it is not surprising that inhibitory neurons can corelease GABA and glycine to optimize postsynaptic control or the feedback regulation of transmission [8]. Indeed, the corelease of glycine and GABA has been confirmed to also exist in the respiratory network [9,10,11]. While GABA/glycine cotransmission seems to be more prevalent in the early stages of development, GABA–glycine cotransmitting neurons (GGCNs) have been identified in adult mice [12]. The relative contribution of the two neurotransmitters in the different parts of the network has, however, not been analyzed yet.

Therefore, we used the photo-stimulation of presynaptic glycinergic neurons expressing channelrhodopsin2 (ChR2) and whole-cell recordings to quantify the relative contributions of glycinergic and GABAergic transmissions between the preBötC and BötC. We found GABA–glycine cotransmission in both directions between the BötC and preBötC, as well as within each region. Interestingly, the relative GABAergic contribution was larger in the preBötC.

## 2. Results

### 2.1. Photo-Stimulation of ChR2 in Glycinergic Neurons

**Figure 1:**
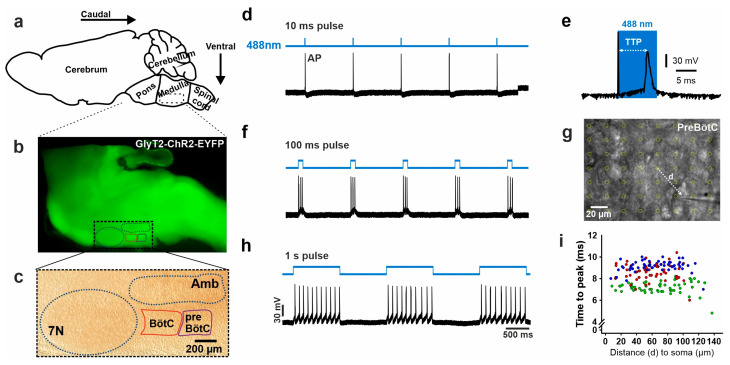
Characterization of neuronal response to poststimulation. Action potential firing upon activation of ChR2. (**a**) Schematic sagittal view of a mouse brain. (**b**) Sagittal slice section of a mouse brainstem expressing ChR2-EYFP and displaying fluorescence in inhibitory neurons with GlyT2 promoter. (**c**) Dodt Gradient Contrast image of the same sagittal slice, showing the fascial nucleus (7N), ambiguous nuclei (AMB), and the area of preBötC and BötC regions. (**d**) Exemplary trace showing an evoked train of AP firing in the patched neuron (upon a series of 10 ms pulses of 488 nm light stimulation (blue trace). (**e**) Enlarged individual AP. An increasing number of APs is recorded in response to increasing pulse durations: (**f**) 100 ms and (**h**) 1 s pulse duration. (**g**) Grid of focus spots (yellow circles) in the preBötC. (**i**) Distribution of the TTPs versus the distance (**d**) from the stimulus (spot) to the patched neuron. The color indicates the response from one neuron (n = 3).

In the first set of experiments, we tested the duration of the stimulus required to ensure that each light pulse only elicits a single action potential. Therefore, neurons were current-clamped and illuminated with light pulses of different durations from 3 ms to 1 s (Figure 1). Recordings show a correlative increase in spike number per pulse when the illumination duration was increased. Repeated stimulation showed that the illumination of 10 ms pulses reliably evokes only a single AP (Figure 1).

Furthermore, we tested whether the time to peak (TTP) of APs (Figure 1), after the onset of light, varies with the distance of light stimulation from a patched neuron. For this purpose, we defined a pattern of point stimulations (Figure 1g) around the soma of the neuron from which APs were recorded. The TTP was 8.3 ± 0.08 ms (n = 3). Interestingly, the delay was rather independent of the distance to the soma (Figure 1i), indicating that the stimulation of the proximal dendrites is sufficient to activate the neuron.

### 2.2. Postsynaptic Responses to Stimulation

**Figure 2:**
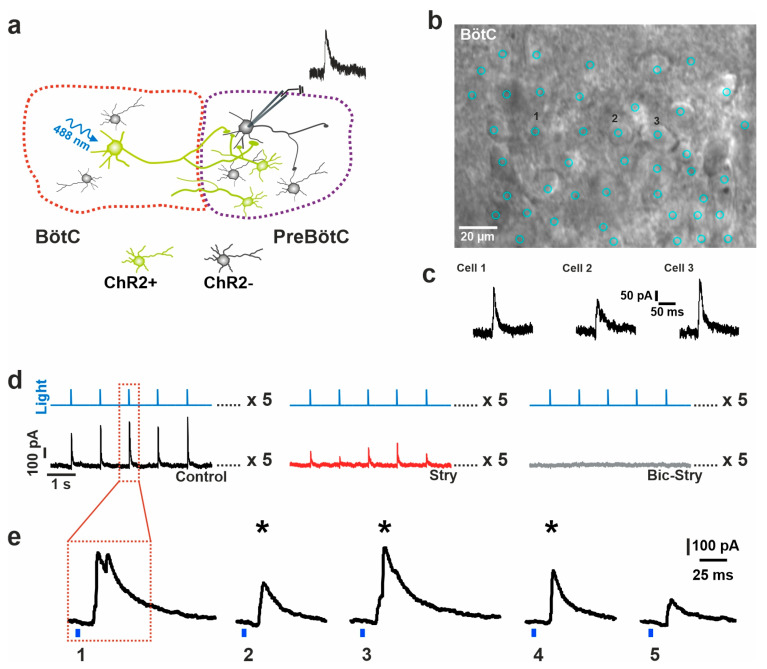
Pharmacological analysis of GABA–glycine cotransmission. (**a**) Schematic drawing of the experimental setup for optogenetic stimulation in the Bötzinger complex (BötC) and simultaneous whole-cell recording in the preBötzinger complex (preBötC). ChR2-expressing neurons in green (ChR2+) and putative excitatory (ChR2-) neurons in gray. (**b**,**c**) Image of the BötC with a pattern of individual illumination spots ((**b**) blue circles). The numbers mark individual cell somata (1–3) that showed a postsynaptic current in response to the stimulation shown in panel c). (**d**) Experimental protocol showing a series of light stimulations corresponding to different locations in a slice (as exemplified in (**b**)). Every pattern is repeated 5 times (control (black), and thereafter repeated under different pharmacological conditions, with strychnine (red) treatment and bicuculline (gray) treatment). (**e**) Examples of evoked IPSCs from 5 repetitive stimulations of the same spot. The three largest eIPSCs that were used for the analysis are marked by an asterisk. Note that eIPSC no. 1 was excluded from the analysis because of its clear biphasic shape (for details of the analysis, see text).

In the next step, we tested whether the stimulation of a presynaptic neuron in the BötC with the parameters defined above is able to reliably elicit evoked IPSCs (eIPSCs) in the preBötC (Figure 2a). First, a stimulating pattern for the point scanning device (UGA-42; see Methods) was defined with the spots aligned to the somata of 15–57 identified presynaptic cells (Figure 2b), while a whole-cell recording of a postsynaptic neuron either in the same region or in the adjacent region was carried out. With this stimulation pattern, the illumination of individual somata resulted in eIPSCs of different amplitudes and shapes (Figure 2c). Each individual spot was then illuminated five times. Usually, each of the five stimuli leads to one eIPSC in the patched postsynaptic neuron (Figure 2d). However, among the five stimuli, double peaks of eIPSCs may sometimes be elicited, in addition to having different amplitudes. Therefore, we selected the three largest single-peak eIPSCs for averaging and further analysis (Figure 2e). 

**Figure 3:**
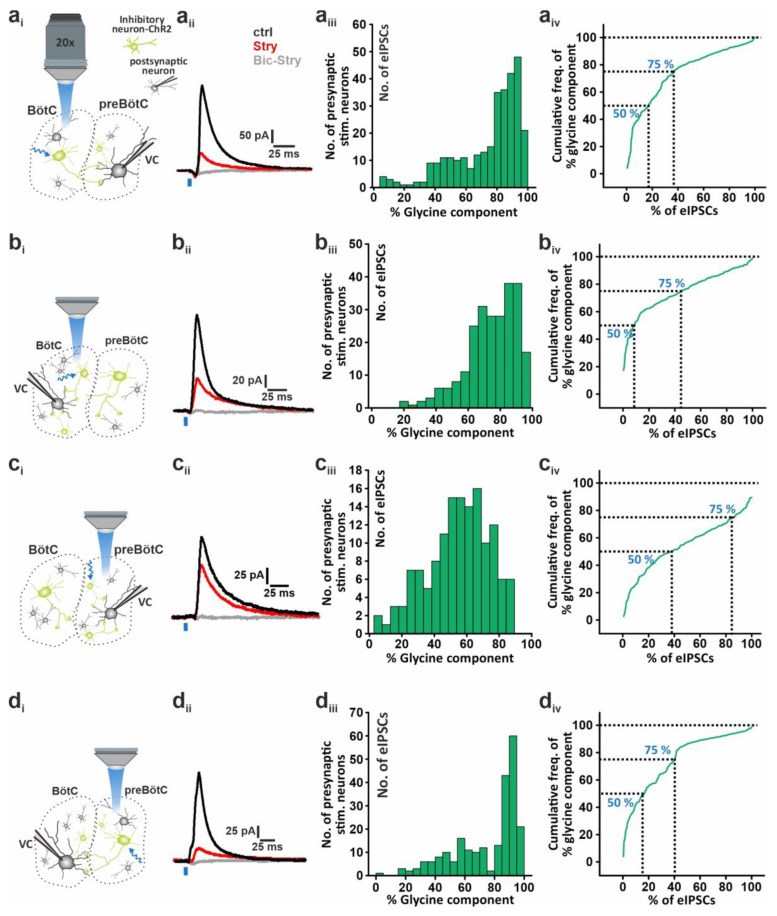
Regional differences in the glycine component of eIPSCs. Experiments were performed in four different configurations (**a**–**d**). (**a**) Stimulation in the BötC, recordings in the preBötC (n = 10 neurons from 7 mice); (**b**) stimulation in the BötC, recordings in the BötC (n = 9 neurons from 8 mice); (**c**) stimulation in the preBötC, recordings in the preBötC (n = 8 neurons from 6 mice); and (**d**) stimulation in the preBötC, recordings in the BötC (n = 11 neurons from 7 mice). The subpanels (**ai**–**di**) show a schematic drawing of the recording configuration. (**aii**–**dii**) Exemplary traces of eIPSCs under control (black): 400 nM strychnine (red) and 20 µM bicuculine (gray) treatment. Stimulus = blue bar. (**aiii**–**diii**) Histograms showing the relative contribution of the glycine current to eIPSCs from individual presynaptic spots (stimulated neurons). (**aiv**–**div**) Cumulative representation of the contribution.

Then, to estimate the relative contribution of GABA and glycine to the eIPSC evoked by a single presynaptic neuron (spot), the same stimulation pattern was repeated in the presence of strychnine (400 nM) and also strychnine + bicuculline (20 µM) to block both components of the fast inhibition (Figure 2d). In case the eIPSC completely disappeared with strychnine, the eIPSC was considered pure glycinergic. If strychnine did not completely block the eIPSC, it was considered a mixed eIPSC. The remaining GABAergic currents, defining the relative GABA contribution, were blocked after the administration of bicuculine (Figure 2d and Figure 3a). The relative glycine component was calculated as follows:

glycine component [%] = 1 − GABA component [%] = eIPSC in Strychnine/eIPSC in control ACSF.(1)

The distribution of the glycinergic contribution was variable among postsynaptic preBötC neurons (n = 10) and presynaptic neurons (305 stimulation sites); however, 64% of the eIPSCs had a high glycine component that was larger than 75% (Figure 3a and Table 1). The median glycine component was 82.2%.

### 2.3. Differential Contribution of Glycine and GABA Transmission within and between BötC and preBötC

These experiments were repeated in the three other configurations: (i) stimulation and recording within the BötC (Figure 3b), (ii) stimulation and recording within the preBötC (Figure 3c), and (iii) stimulation in the preBötC and recording in the BötC (Figure 3d). 

The distribution of the glycine component for (i) stimulation and recording within the BötC was also predominantly glycinergic with a median of 76.9% (Figure 3b). Interestingly, for stimulation and recording within the preBötC (ii), a larger contribution of GABAergic currents of 43.1% (median glycine component: 56.9%; Figure 3c) was observed. When the preBötC was stimulated and the recording electrode was in the BötC, the median of the glycine component was 86.5%, the largest value of all four configurations (Figure 3d). 

**Figure 4:**
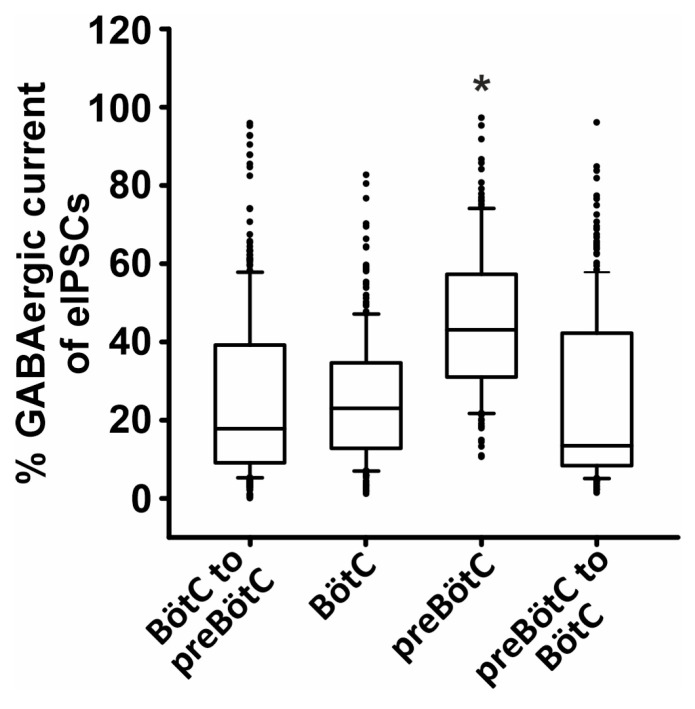
Summary data of % of GABAergic contribution in eIPSCs in the four different configurations shown in Figure 3. GABAergic contribution was significantly higher in the case of transmission within preBötC compared to the other three transmissions. The line inside box plots represents the median of each distribution. Black dots represent individual data points. BötC to preBötC, 10 neurons (305 presynaptic stimulation sites); BötC to BötC, 11 neurons (244 presynaptic stimulation sites); preBötC to preBötC, 8 neurons (141 presynaptic stimulation sites); and preBötC to BötC, 9 neurons (233 presynaptic stimulation sites).

Taking the GABAergic contributions in all four recording configurations, statistical analysis shows a significantly higher GABA transmission within the preBötC compared to the other three configurations (medians of GABA contribution: BötC to preBötC, 17.8%; within BötC, 23.1%; within preBötC, 43.1%; and preBötC to BötC, 13.5%; one-way ANOVA on ranks: df = 3, *p* < 0.001; Figure 4). 

In addition, looking at the 75% cutoffs of glycine contributions in an eIPSC, we see again that within the preBötC there is an obvious decrease in the percentage of total eIPSCs with ≥75% glycinergic contribution compared to the other configurations (Table 1, Figure 3 aiv, biv, civ, div), indicating a higher amount of GABAergic transmission within this region. Of note is the fact that glycine and GABA corelease dominated most eIPSCs in all configurations with percentages ranging between 90.4% and 100% of total eIPSCs (corelease: BötC to preBötC, 90.4%; within BötC, 95.9%; within preBötC, 100%; and preBötC to BötC, 91%). Corelease was considered when less or equal to 95% of an eIPSC was glycinergic. Our results show a dominance of corelease in the BötC and preBötC during the early postnatal stage.

### 2.4. Glycinergic vs. Non-Glycinergic Postsynaptic Neurons

**Figure 5:**
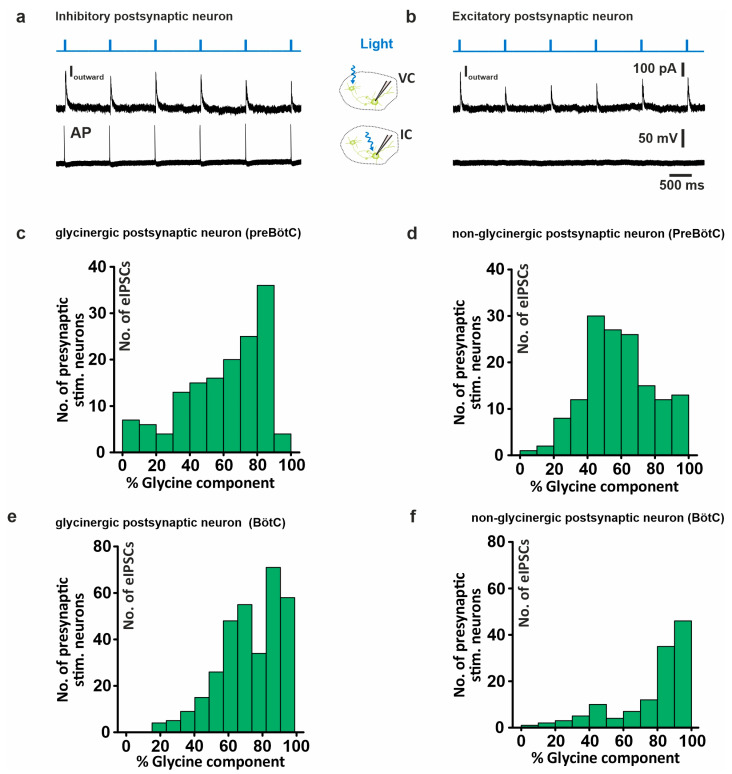
Differential contribution of glycinergic current in glycinergic and non-glycinergic postsynaptic neurons. (**a**) Example of light-evoked IPSCs (upper black trace), and voltage trace of current-clamped glycinergic neuron (expressing ChR2) showing AP firing (lower black trace). (**b**) Example of light-evoked IPSCs (upper black trace), and voltage trace of current-clamped excitatory neuron (not expressing ChR2) (lower black trace). (**c**) Histogram showing the distribution of the percentage contribution of the glycine current of eIPSCs in a glycinergic postsynaptic neuron in preBötC upon the illumination of presynaptic ChR2-positive glycinergic neurons. (**d**) Histogram as in (**c**) but in non-glycinergic postsynaptic neurons in preBötC. (**e**) Histogram as in (**c**) but in BötC. (**f**) Histogram as in (**d**) but in BötC (non-glycinergic postsynaptic neurons in BötC, n = 5 (no. of eIPSCs = 125); inhibitory postsynaptic neurons in BötC (no. of eIPSCs = 325), n = 14; non-glycinergic postsynaptic neurons in preBötC, n = 7 (no. of eIPSCs = 146); inhibitory postsynaptic neurons in preBötC, n = 7 (no. of eIPSCs = 146)).

The analysis above did not consider the type of postsynaptic neuron. In a subset of recordings, however, we collected information about the postsynaptic neuron (Figure 5): Neurons that fired APs when directly illuminating the neuron in the current clamp mode were identified as glycinergic neurons. If no AP was elicited, the postsynaptic neuron was considered non-glycinergic. This group of neurons is rather heterogeneous, comprising excitatory neurons but potentially also including inhibitory neurons that did not develop from GlyT2-expressing precursors [10,12]. Without taking the region of stimulation into consideration, the distribution histogram of the glycine component of eIPSCs recorded from non-glycinergic postsynaptic neurons (Figure 5d) in the preBötC appeared to be left-shifted as compared to the histogram of glycinergic neurons (Figure 5c), indicating a larger GABAergic component in the non-glycinergic postsynaptic neurons of the preBötC. Although the statistical comparison of the glycine component of all eIPSCs did not show a significant difference between non-glycinergic (median 57.1%, n = 146) and glycinergic neurons (67,9%, n = 146; Mann–Whitney U rank sum test; *p* = 0.094), a comparison of the two distributions using a G-test of goodness of fit revealed a significant difference (G = 73.324, df = 9, *p*-value = 3.383 × 10^−12^). A comparison of the BötC neurons (Figure 5e,f) revealed a significant shift of the histogram towards a larger GABAergic component in the glycinergic neurons (G = 51.179, df = 9, *p*-value = 6.46 × 10^−8^). Here, the relative glycine component of all eIPSCs was significantly larger in the non-glycinergic (median 86.0%, n = 125) compared to the glycinergic neurons (73.8%, n = 325; Mann–Whitney U rank sum test; *p* < 0.01). Overall, the GABAergic input to non-glycinergic and glycinergic neurons was significantly higher in the preBötC than in the BötC (one-way ANOVA on ranks: df = 3, *p* < 0.001).

## 3. Discussion

The optogenetic stimulation of glycinergic neurons in sagittal brainstem slices revealed two major results. First, GABA–glycine cotransmission is the dominant mode in both preBötC and BötC during the early postnatal stage. Second, BötC and preBötC are interconnected bidirectionally through GGCNs. 

### 3.1. Differential Loading of Synaptic Vesicles 

Currently, it is unknown to what degree glycine and GABA are loaded (i) into the same vesicle or (ii) into different vesicles in the same neuron. If GABA and glycine were always loaded together into all vesicles, one would expect that 100% of miniature inhibitory currents (mIPSCs) would be mixed events. However, this is not the case. From an earlier set of experiments, we know that only 20–40% of mIPSCs in the preBötC result from GABA/glycine corelease [9]. Thus, the current observation favors the model showing that glycine and GABA are mostly loaded into different synaptic vesicles.

### 3.2. GABA/Glycine Cotransmission Is the Default Mode in the Neonatal Respiratory Network 

Our data clearly show that GABA/glycine cotransmission is the default mode in the neonatal respiratory network. Although GABA/glycine cotransmission has been postulated for many brainstem regions [8,9,13,14,15,16], our study is the first that compares GABA/glycine cotransmission in different circuit motifs on a functional level. Interestingly, we found that the GABAergic eIPSC component was larger when the recording and stimulation were performed within the preBötC. In the preBötC, the GABAergic component was the largest in non-glycinergic neurons, which can be assumed to be, at least to a large extent, excitatory neurons. This is in line with findings from Ritter and Zhang, suggesting an important role of GABAAR in the modulation of respiratory rhythmogenesis [17]. The difference between the preBötC and BötC is probably not due to a generally different distribution of GGCNs in the two regions, since the number of GGCNs in the two regions was not different (Supplementary Figure S1). Since GABA can be synthesized from glutamate by two isoforms of the glutamate-decarboxylase, it remains possible that the utilization of two types of glutamate decarboxylase, GAD1 (GAD67) and GAD2 (GAD65), is different in neurons targeting the two regions. Also, a potential difference in the postsynaptic expression of GABA and glycine receptors needs to be tested in the future. Nevertheless, almost all stimulations led to a glycinergic current, which was not surprising since the glycine transporter 2 (GlyT2) was used for driving the Cre-recombinase expression. This is also in line with previous studies in the VRC [7] and the generally high levels of glycine in the brainstem [18].

### 3.3. Limitations 

Although synaptic events could be readily induced by pointing the laser to the soma of a visually identified neuron, we cannot exclude that in some cases a dendritic/axonal process of a neighboring neuron was stimulated. This is due to the expression of channelrhodopsin (ChR2(H134R)/EYFP) along the entire plasma membrane. Moreover, the stimulation of the somata sometimes resulted in the simultaneous activation of neighboring neurons, leading to eIPSCs with two peaks (Figure 2). Although we eliminated this type of response from our analysis, we cannot completely rule out that cotransmission events are due to the simultaneous activation of two neurons (with very similar conduction velocity stimulated at the same distance of the recorded neuron). If one of these neurons was purely GABAergic and the other was purely glycinergic, a false-positive detection of cotransmission is indeed possible. During embryonic development, most if not all inhibitory neurons are expected to corelease glycine and GABA [10]. However, based on the genetic fate mapping using a mouse line that expresses tdTomato in neurons with the simultaneous activation of the GAD65 and Glyt2 promoter (COTRANS mice [12]), one would expect that approximately 18% of the neurons that express ChR2 should be purely GABAergic in the first postnatal week [12]. 

### 3.4. Implications for Network Function 

It has been shown that the activation of glycinergic neuron stimulation can modulate respiratory network activity in accordance with traditional central pattern generator (CPG) models [19,20,21]. However, through recordings from optogenetically identified glycinergic neurons, we discovered that these neurons do not strictly follow the bursting patterns that would be predicted by the CPG models [22]. Moreover, neurons that are activated during respiratory phase transitions are also found outside the BötC and preBötC [23]. Furthermore, differential effects observed after the blockade of glycine receptors or GABA [24,25] are difficult to explain using the CGP models. Given the widespread nature of glycine/GABA cotransmission observed in our present study, these previous discrepancies might be related to postsynaptic receptor expression more than the presynaptic inhibitory neuron phenotype. This aspect needs to be addressed in future studies.

## 4. Materials and Methods

### 4.1. Animals 

This study was carried out in accordance with the ARRIVE guidelines, with the guidelines for the welfare of experimental animals issued by the European Communities Council Directive 2010/63/EU, and with the German Protection of Animals Act (TierSchG). All experiments and animal care were conducted in agreement with § 4 Abs. 3 TierSchG and were approved and registered (T12/11) by the animal welfare office and commission of the University Medical Center Göttingen. The mouse line expressing channelrhodopsin2 (ChR2–EYFP) was generated from crossbreeding mice expressing Cre-recombinase under the control of glycine transporter 2 promoters (Tg(Slc6a5icre)121Veul) with mice expressing ChR2 in a Cre-dependent manner, 129S-Gt(ROSA)26Sortm32(CAG-COP4*H134R/EYFP)Hze/J mice [19]. To identify glycinergic and GABAergic neurons, we used the COFLUOR mouse line Tg(Gad2-tdTomato)DJhi × Tg(Slc6a5- EGFP)1Uze. This line expresses EGFP under the control of the GlyT2 promotor in glycinergic neurons and tdTomato under the control of the GAD65 promotor in GABAergic neurons. GGCNs appear yellow in the fluorescent microscope [12,26]. 

### 4.2. Acute Brainstem Slice Preparation

Mice between postnatal day 2 (P2) and 7 (P7) were anesthetized with isoflurane and decapitated, and the brain was isolated in ice-cold oxygenated artificial cerebrospinal fluid (ACSF) containing 118 mM NaCl, 25 mM NaHCO_3_, 1 mM NaH_2_PO_4_, 1.5 mM CaCl_2_, 3 mM KCl, 1 mM MgCl_2_, and 30 mM D-glucose. The brainstem was separated from the remaining brain and fixed with cyan acrylate glue on an agar block with its rostral and caudal ends directed horizontally for sagittal sectioning (Figure 1). Per mouse, two 250 µm thick sagittal brainstem slices, each containing both the BötC and preBötC regions, were obtained using a vibratome (VT1200S or VT1000S, Leica, Wetzlar, Germany) and collected in oxygenated ACSF at room temperature, allowing for a minimum recovery period of 30 min before starting the experiment. 

### 4.3. Electrophysiology 

Slices were transferred to a recording chamber on an upright epifluorescence microscope Examiner.Z1 (Zeiss, Oberkochen, Germany), which was held stable with a slice anchor (Warner Instruments, Holliston, MA, USA) and superfused continuously with oxygenated ACSF at a temperature of ~30 °C with a custom-made heat-exchanger, which was controlled by an SC100 immersion circulator (Thermo Scientific, Waltham, MA, USA) and driven by a peristaltic pump (Watson-Marlow, Rommerskirchen, Germany). For the detection of EYFP fluorescence (ChR2-EYFP), slices were illuminated by a SOLA SE light engine (Lumencor, Beaverton, OR, USA) using a filter set consisting of a BP 450–490 nm for excitation, a beam splitter (BS) at 495 nm, and a 500–550 nm emission bandpass (BP) filter (Filter set 38; Zeiss, Oberkochen, Germany). Images were captured via a CCD camera (Sensicam; PCO, Kelheim, Germany) controlled by the open-source imaging software micro-manager (micro-manager.org) [27]. Patch electrodes were pulled from borosilicate glass capillaries (Science Products, Hofheim am Taunus, Germany) using a DMZ universal electrode puller (Zeitz-Instrumente, Martinsried, Germany) or PP-830 (Narishige, London, UK). We used electrodes with resistance between 3 and 7 MΩ when filled with intracellular solution (see below). For patching neurons in the brainstem, patch pipettes were filled with intracellular solution containing 140 mM K-Gluconic acid, 1 mM CaCl_2_, 2 mM MgCl_2_, 4 mM Na2ATP, 10 mM EGTA, and 10 mM HEPES. Whole-cell patch-clamp recordings were accomplished in voltage clamp (VC) and current clamp (IC) modes using a MultiClamp 700A patch-clamp amplifier (Molecular Devices; San Jose, CA, USA), and data were acquired with DigiData 1322A (Molecular Devices; San Jose, CA, USA). For recording inhibitory synaptic currents, membrane potential was clamped to 0 mV, and evoked inhibitory synaptic currents (eIPSCs) were recorded as outward currents given the equilibrium potential of chloride (E[Cl-]) −77 mV. To discriminate between glycinergic receptor (GlyR)-mediated currents and gamma aminobutyric acid receptor (GABA_A_R)-mediated currents, strychnine (400 nM; Sigma-Aldrich Co., St Louis MO, USA) was applied to block GlyR-mediated currents. To confirm that the remaining currents were GABA_A_R-mediated, bicuculline (20 µM; Sigma-Aldrich Co., St Louis MO, USA) was added to the strychnine-containing solution. 

### 4.4. Optogenetic Stimulation

The stimulation of the light-sensitive ChR2 expressed in glycinergic neurons was accomplished with a point scanning device for localized photomanipulation (UGA-42 Firefly, Rapp Optoelectronics (ROE), Hamburg, Germany) and ROE SysCon software (Version 1.2.0.7; Rapp Optoelectronics, Hamburg, Germany). The UGA-42 Firefly system delivers a single light spot at a time that moves to a coordinate to illuminate, e.g., individual neurons in the tissue slice (Figure 1). For illumination, we used a 488 nm CW solid-state laser (Sapphire; Coherent, Göttingen, Germany), which was coupled into the UGA-42 via a 3.5 µm 0.13 NA single-mode fiber (Thorlabs Inc., Newton, NJ, USA) and controlled via an acousto-optical modulator (AOM; MT110-A1-VIS; AA OPTO-ELECTRONIC, Orsay, France). Digital control of the AOM was realized using a custom-made AOM controller and TTL signals from the UGA-42. The resulting light spot using a 1.0 NA 20× PLAN-apochromat objective (Zeiss, Oberkochen, Germany) had a full width at half maximum intensity (FWHM) of 6.6 µm, which is small enough to hit a single presynaptic neuron. The power was kept between 9 and 11 mW to allow for the reliable depolarization of presynaptic neurons. Neurons were current-clamped and illuminated with light pulse durations of up to 1 s. 

### 4.5. Data Handling and Statistical Analysis

Electrophysiological data were stored as axon binary files (ABF) and analyzed with Clampfit 10 (Molecular Devices; San Jose, CA, USA) or LabChart8 (ADInstruments, Oxford, UK). Prior to eIPSC peak detection, a baseline correction was performed in Clampfit 10 software by averaging 20 ms of the recording before the optical stimulation and subtracting the value from each point of the recording. Next, the detection threshold was set to the highest peak of the baseline; thus, signals under (or equal to) this threshold were rejected. Statistical graphs and tests were performed with the software SigmaPlot 12.5 (Systat Software GmbH, Erkrath, Germany) or R (www.r-project.org) [28,29].

## 5. Conclusions

Taken together, we could show a regional heterogeneity between the preBötC and BötC and between inhibitory neurons and excitatory neurons regarding the distribution of the GABAergic and glycinergic inputs. Furthermore, our data point towards a high degree of redundancy of GABAergic and glycinergic transmission mediated mainly by the differential loading of GABA and glycine into presynaptic vesicles. The data presented here describe the situation in the neonatal network. Future studies have to be conducted to reveal the situation in the adult mouse. 

## Figures and Tables

**Table 1:** Percentage of total eIPSCs with ≥50% and ≥75% glycinergic contribution.

	BötC to preBötC	Within BötC	Within preBötC	preBötC to BötC
≥50% glycinergic	83%	92%	63%	83%
≥75% glycinergic	64%	55%	15%	60%

## Data Availability

The original contributions presented in the study are included in the article/Supplementary Materials; further inquiries can be directed to the corresponding author/s.

## Supplementary Materials

The supporting information can be downloaded at https://www.mdpi.com/article/10.3390/ijms25063128/s1. References [30,31] are cited in Supplementary Materials.
