# Supplementary material for: The Relative Contribution of Glycine–GABA Cotransmission in the Core of the Respiratory Network
Source: Int J Mol Sci. 2024 Mar 8;25(6):3128. doi: 10.3390/ijms25063128 (PMC10970536; doi:10.3390/ijms25063128)
Supplement: Supplementary file 1 [file ijms-25-03128-s001.zip › ijms-2894977-supplementary.pdf]

Supplementary material

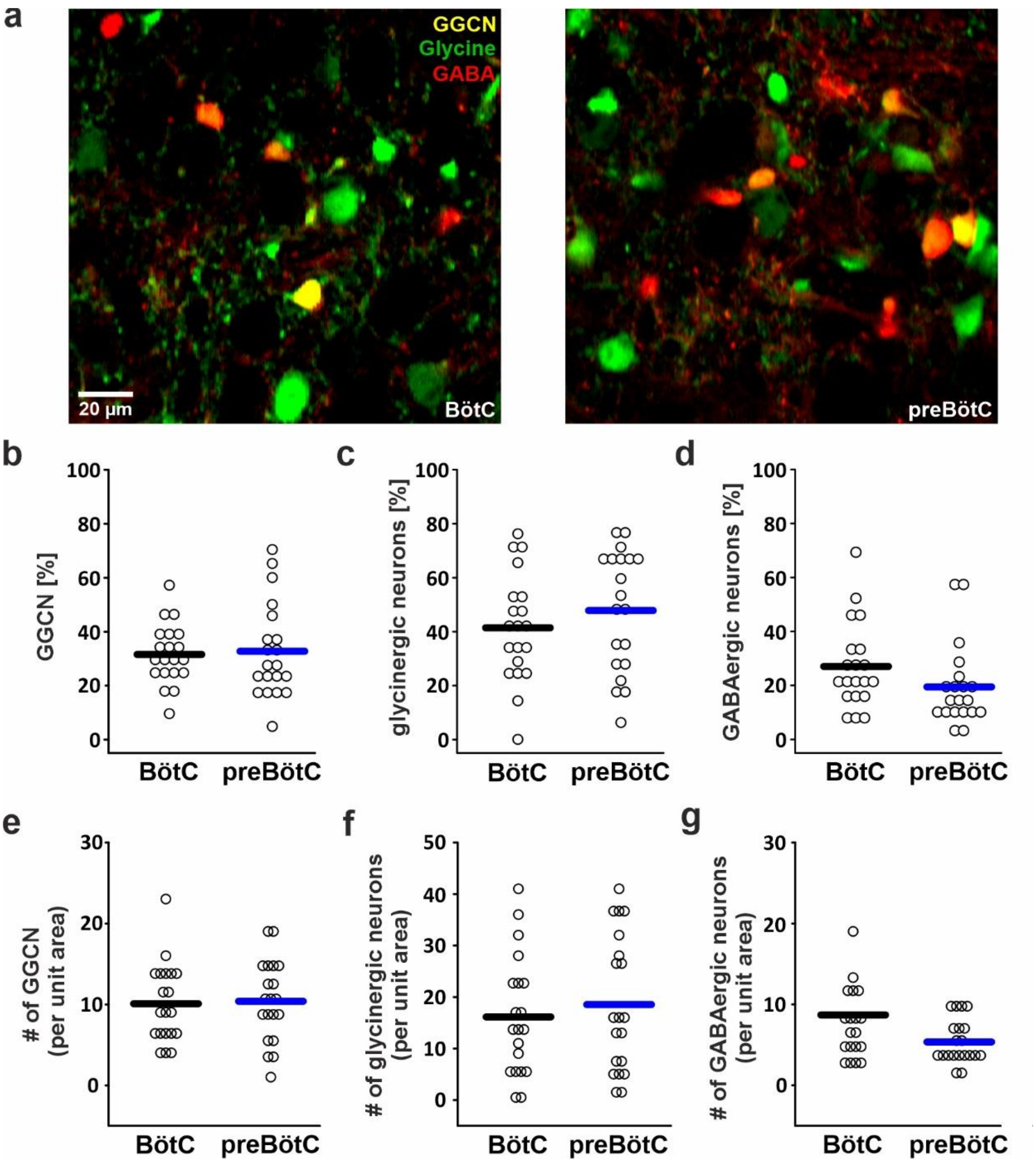

Supplementary figure S1: BötC and preBötC show similar distribution of GABA-glycine cotransmitting neurons (GGCNs). a) Merged two photon images of BötC (left) and preBötC (right) in sagittal brainstem slices from COFLUOR mice expressing EGFP in glycinergic and tdTomato in GABAergic interneurons. B-d) Data plot of percentage GGCNS (b), glycinergic neurons (c), and GABAergic neurons (d) in BötC and preBötC regions, respectively. e-g) number of neurons per optical plane; number of GGCN (e), glycinergic neurons (f), and GABAergic neurons (g) in BötC and preBötC

regions, respectively (5 slices, 4 mice, 4 stacks for each slice). No significant difference between BötC and preBötC was found.

Supplementary methods: 2-Photon imaging of COFLUOR acute brainstem slices

Imaging of COFLUOR acute brainstem tissues to detect glycinergic and GABAergic neurons in BötC and preBötC was performed with a 2-Photon (2P) laser-scanning microscope (TriMScope; LaVision BioTec). A 20x (1.0 NA) water immersion objective lens (Zeiss, Oberkochen, Germany) and GaAsP photomultipliers for non-descanned detection (Hamamatsu Photonics K.K., Hamamatsu, Japan) was used, and two photon excitation was achieved with a Ti:Sapphire Laser (MaiTai BB, SpectraPhysics, Santa Clara CA, USA). COFLUOR slices were excited with three wavelengths: 720, 800, and 900 nm, and emission was detected through the three band-pass emission filters: 641/75, 531/40, and 475/50 nm. “Imspector” images generated with 2-Photon microscopy were exported as TIFF format and analyzed in ImageJ. To avoid errors from spectral overlapping of EGFP and tdTomato, we processed the images using a spectral unmixing plug-in in ImageJ to separate the signals of the two fluorophores using non-negative tensor factorization [1, 2].

References

1. Neher, R. A.; Mitkovski, M.; Kirchhoff, F.; Neher, E.; Theis, F. J.; Zeug, A., Blind source separation techniques for the decomposition of multiply labeled fluorescence images. *Biophys J* **2009,** 96, (9), 3791-800.
2. Oke, Y.; Miwakeichi, F.; Oku, Y.; Hirrlinger, J.; Hulsmann, S., Cell types and synchronous-activity patterns of inspiratory neurons in the preBotzinger complex of mouse medullary slices during early postnatal development. *Sci Rep* **2023,** 13, (1), 586.
